# GdbMTB: a manually curated genomic database of magnetotactic bacteria

**DOI:** 10.1093/database/baaf090

**Published:** 2026-01-15

**Authors:** Runjia Ji, Yongxin Pan, Wei Lin

**Affiliations:** Key Laboratory of Planetary Science and Frontier Technology, Institute of Geology and Geophysics, Chinese Academy of Sciences, No. 19, Beitucheng Western Road, Chaoyang District, Beijing 100029, China; College of Earth and Planetary Sciences, University of Chinese Academy of Sciences, No. 19A, Yuquan Road, Shijingshan District, Beijing 100049, China; Key Laboratory of Planetary Science and Frontier Technology, Institute of Geology and Geophysics, Chinese Academy of Sciences, No. 19, Beitucheng Western Road, Chaoyang District, Beijing 100029, China; College of Earth and Planetary Sciences, University of Chinese Academy of Sciences, No. 19A, Yuquan Road, Shijingshan District, Beijing 100049, China; Key Laboratory of Planetary Science and Frontier Technology, Institute of Geology and Geophysics, Chinese Academy of Sciences, No. 19, Beitucheng Western Road, Chaoyang District, Beijing 100029, China; College of Earth and Planetary Sciences, University of Chinese Academy of Sciences, No. 19A, Yuquan Road, Shijingshan District, Beijing 100049, China

## Abstract

Magnetotactic bacteria (MTB) are a unique group of microorganisms capable of navigating along geomagnetic field lines through the biomineralization of intracellular magnetic nanocrystals called magnetosomes. While genomic analyses have substantially advanced our understanding of these predominantly uncultured microorganisms, MTB genomic data remain scattered across multiple databases with inconsistent quality profiles and incomplete metadata, limiting comprehensive research efforts. To address these challenges, we developed the Genomic Database of Magnetotactic Bacteria (GdbMTB), the first comprehensive, curated genomic resource dedicated to MTB. The current version of GdbMTB integrates 365 publicly available MTB genomes and their associated metadata. Through a standardized bioinformatics workflow, it provides detailed genome quality assessments, taxonomic classifications, and annotations of magnetosome biomineralization genes, ensuring reliable data for downstream analyses. The curated metadata, encompassing environmental context and publication details, offers crucial research background, enabling users to trace the provenance of each genome. Additionally, GdbMTB offers a suite of bioinformatics tools and an analysis pipeline to facilitate advanced MTB studies. GdbMTB enhances accessibility to MTB genomic data, thereby promoting interdisciplinary research in microbiology, geomicrobiology, and biomineralization studies.

Database URL: https://www.gdbmtb.cn/

## Introduction

Magnetotactic bacteria (MTB) are microorganisms capable of sensing and navigating along the geomagnetic field lines to locate optimal niches in aquatic environments [1–4]. As the earliest known magnetoreceptive organisms, MTB provide crucial insights into the origin and mechanisms of microbial magnetoreception [5, 6]. Their specialized magnetic organelles, termed magnetosomes, are membrane-bound nano-sized crystals of magnetite (Fe_3_O_4_) and/or greigite (Fe_3_S_4_) [7–9]. Magnetosomes serve as exemplary models of biologically controlled biomineralization, providing valuable perspectives on the origin and evolution of bacterial organelles. Following the death of MTB cells, magnetosomes can be preserved in sediments as magnetofossils, serving as important proxies for paleoenvironmental and paleomagnetism reconstruction [10].

MTB exhibit remarkable ecological versatility, thriving in diverse aquatic environments ranging from freshwater to extreme habitats [11, 12]. Their resilience under extreme conditions makes them particularly valuable as model organisms for astrobiological studies [12]. Through magnetosome biomineralization, MTB accumulate significantly higher intracellular iron levels than other microorganisms [13]. Moreover, MTB exhibit remarkable metabolic versatility and contain various intracellular inclusions, including sulfur globules [14], polyhydroxybutyrate [15], selenium granules [16], polyphosphate [17], Ca/Mg-rich polyphosphate granules [18], calcium phosphate [19], calcium carbonate [20], ferrosomes [21], silica globules [22], and polyhydroxyalkanoates [23].

Despite their identification across at least 16 bacterial phyla, the majority of MTB remain uncultured, limiting comprehensive analyses of their metabolic capacities and ecological functions [11]. However, advances in high-throughput sequencing technologies, coupled with the development of metagenomics and single-cell genomics, have enabled the recovery of numerous MTB genomes from environmental samples [6, 24–28]. The molecular genetic basis of magnetotaxis is the magnetosome gene clusters (MGCs), which include genes necessary for magnetosome biosynthesis and arrangement [28, 29]. Comparative genomic analyses focusing on the MGCs and corresponding magnetosome morphotypes have inferred biosynthesis mechanisms among the major MTB-containing lineages, specifically *Pseudomonadota* (previously known as the *Proteobacteria*), *Desulfobacterota* (previously known as the *Deltaproteobacteria*), and *Nitrospirota* (previously known as the *Nitrospirae*) [30, 31]. Genome-based metabolic analysis has additionally been used to elucidate their ecological function within varied environments [18, 27, 32–36]. Collectively, these genome-based studies have significantly enhanced our understanding of MTB and magnetotaxis from phylogenetic, ecological, and evolutionary perspectives.

A significant challenge in MTB and magnetotaxis research lies in the dispersed nature of publicly available genomic data across multiple databases, which are often characterized by inconsistent quality and incomplete metadata. Crucial contextual information, such as geographic locations and environmental physicochemical parameters, remains largely unorganized, hindering downstream integrative analyses. To address these challenges, we present the Genomic Database of Magnetotactic Bacteria (GdbMTB) (https://www.gdbmtb.cn/), a comprehensive, curated resource consolidating 365 publicly available MTB genomes and their associated metadata as of June 2024. As the first dedicated MTB genomic database, GdbMTB provides a valuable platform for systematic MTB research, facilitating comparative analyses and interdisciplinary investigations.

## Materials and methods

### Core data and metadata collection

A total of 365 publicly available MTB genomes were downloaded from the NCBI Genome database [37], the eLibrary of Microbial Systematics and Genomics database (eLMSG) [38], the Integrated Microbial Genomes database (IMG database) [39], the European Nucleotide Archive [40], and the RAST Server [41]. Then, all genomes were named using the respective database accession plus the organism’s name. 296 MTB genomes were downloaded using the ncbi-datasets-cli v14.4.0 [42] from the NCBI Genome database along with the genome reports using ‘download’ and ‘summary’ commands, respectively. MTB genomes downloaded from NCBI were renamed using the GenBank accession (starting with ‘GCA_’) plus the organism’s name. 63 MTB genomes were downloaded from the eLMSG database and renamed using the eLMSG accession (starting with ‘LMSG_’) plus the organism’s name. Four MTB genomes were downloaded from the IMG database (IMG Genome IDs: 2264265205, 2651870060, 3300015153, and 3300022116) and renamed with a prefix ‘IMG_’ plus the genome ID and organism’s name. One genome was downloaded from the RAST server and renamed using the RAST ID with a prefix ‘RAST_’, and another from ENA, renamed with the sequence accession plus the organism’s name.

Sample metadata of all genomes, including sampling information (such as geographic coordinates and locations), were obtained by searching and checking the associated original publications and the NCBI BioSample records. Habitats were classified into 11 categories based on sampling site characteristics (e.g. depth, area) and physicochemical properties (e.g. oxygen concentration, salinity, temperature, pH). These categories include freshwater, brackish, marine, marine oxygen minimum zone (OMZ), bog/peatland (freshwater), bog/peatland (brackish), bog/peatland (acidic), hypersaline, thermal environment (hot spring water), thermal environment (hydrothermal vent chimney), and subsurface/deep subsurface. Note that an area is categorized as ‘brackish’ either when it is explicitly described as such in the original publication or when the reported salinity ranges from 0.5 to 35 parts per thousand (ppt). This classification includes inland salt lakes and estuarine systems.

Genome metadata encompasses detailed information related to the submission of genomes to public databases. This includes various accession identifiers linked to the genome, such as GenBank, BioProject, BioSample, and WGS entries, along with submission details such as the sequencing platform, submitter’s name, submitting institution, and submission date. For genomes downloaded from NCBI, the genome reports were first obtained using the ncbi-datasets-cli v14.4.0 [42] with the ‘summary’ command. Genome metadata were then resolved from the genome reports using a custom Python script, retrieve_metadata_from_ncbi_assembly_reports_json.py. Genome metadata of MTB genomes from other databases were manually searched, collected, and verified.

Reference metadata provides details about publications associated with the genomes, including the names of the authors, the journals, the article titles, the publication dates, as well as identifiers such as PMIDs and DOIs. For genomes downloaded from NCBI, reference metadata was obtained using the Entrez Programming Utilities (E-utilities) provided by NCBI, with the Bio.Entrez module from the Biopython library [43]. Steps include: (1) Entrez.esearch was used to search the NCBI Genome database with unique accession numbers. (2) Entrez.elink was used to retrieve PubMed IDs (PMIDs) of the associated publications. (3) Entrez.efetch was used to retrieve reference metadata in XML format, including titles, publication dates, DOIs, authors, and journal names. Following the approaches described above, a custom Python script (use_ncbi_entrez_api_get_reference.py) was developed to automate this process. In contrast, reference metadata of MTB genomes from other databases were manually searched, collected, and verified.

### Genome quality assessment

Genome completeness and potential contamination were quantified using established tools, including CheckM v1.2.2 [44], CheckM2 v1.0.1 [45], BUSCO v5.6.1 [46], and GUNC v1.0.6 [47]. General genomic features, including genome size (bp), GC content (%), number of contigs, and N50 (bp), were evaluated with QUAST v5.0.2 [48]. Coding sequences (CDS) were predicted using Prodigal v2.6.3 [49]. The ribosomal RNA (rRNA) genes were identified using rRNAFinder v1.1.1 (https://github.com/xiaoli-dong/rRNAFinder). The transfer RNA (tRNA) genes were detected using Aragorn v1.2.41 [50]. For each genome, a quality label was attached based on CheckM-estimated completeness and contamination [44], and following the standards for minimum information about a single amplified genome (MISAG) and minimum information about a metagenome-assembled genome (MIMAG) [51].

### MTB taxonomic classification and phylogenetic analysis

MTB genomes were taxonomically assigned using GTDB-tk v2.4.0 [52] with the ‘classify_wf’ workflow based on the Genome Taxonomy database release 220 [53]. MTB genomes were then dereplicated at the species level with dRep v3.5.0 [54] using the 95% average nucleotide identity (ANI) species-delineation threshold, which resulted in 252 unique species. For each species, a representative genome was selected based on genomic quality and used to construct ‘the Tree of MTB’ alongside 278 closely related non-MTB GTDB representative genomes. The 120 single-copy marker proteins [55] in these genomes were first identified using GTDB-Tk v2.4.0 [52] ‘identify’ module. Concatenated multiple sequence alignment was then generated using ‘align’ module. The phylogenomic tree was constructed using IQ-TREE v2.0.3 [56] with a ‘TEST’ option for optimal substitution model selection (LG + F + I + G4) and ultrafast bootstrap value set to 1000. The resulting tree was then visualized using iTOL v6 [57].

### MGC annotation and representative MGC depiction

To annotate magnetosome genes within MTB genomes efficiently, MagCluster v0.2.11 [58] was used for genome annotation, utilizing a published magnetosome protein dataset as annotation reference. The MGCs were then screened and visualized. Representative MGCs at the species level were manually checked and displayed on the ‘Browser-Magnetosome gene clusters’ page of GdbMTB website.

### Website development

The GdbMTB website was developed using Python 3 and the Flask web framework. The front end was built with HTML, CSS, JavaScript, and Bootstrap v5.1.3 (https://getbootstrap.com/). The website is deployed on the PythonAnywhere server (https://www.pythonanywhere.com/) and is accessible at https://www.gdbmtb.cn/. Compatibility testing confirmed that the website functions on major browsers, including Chrome, Microsoft Edge, Firefox, and Safari.

### Data processing and visualization

Data processing and visualization were conducted within the JupyterLab v4.2.1 workspace (https://jupyter.org/). Data organization was performed using pandas v2.2.2 (https://pandas.pydata.org/), while visualizations were created with matplotlib v3.8.4 (https://matplotlib.org/), seaborn v0.13.2 (https://seaborn.pydata.org/), Vega-Altair v5.3.0 (https://altair-viz.github.io/), plotly v5.23.0 (https://github.com/plotly/plotly.py), and manim v0.18.1 (https://www.manim.community/). Final adjustments to figures were made using the open-source vector graphics editor Inkscape (https://inkscape.org/). The bioinformatics pipeline diagram was created using Whimsical (https://whimsical.com/).

## Results and discussion

### Description of GdbMTB

**Figure 1.**
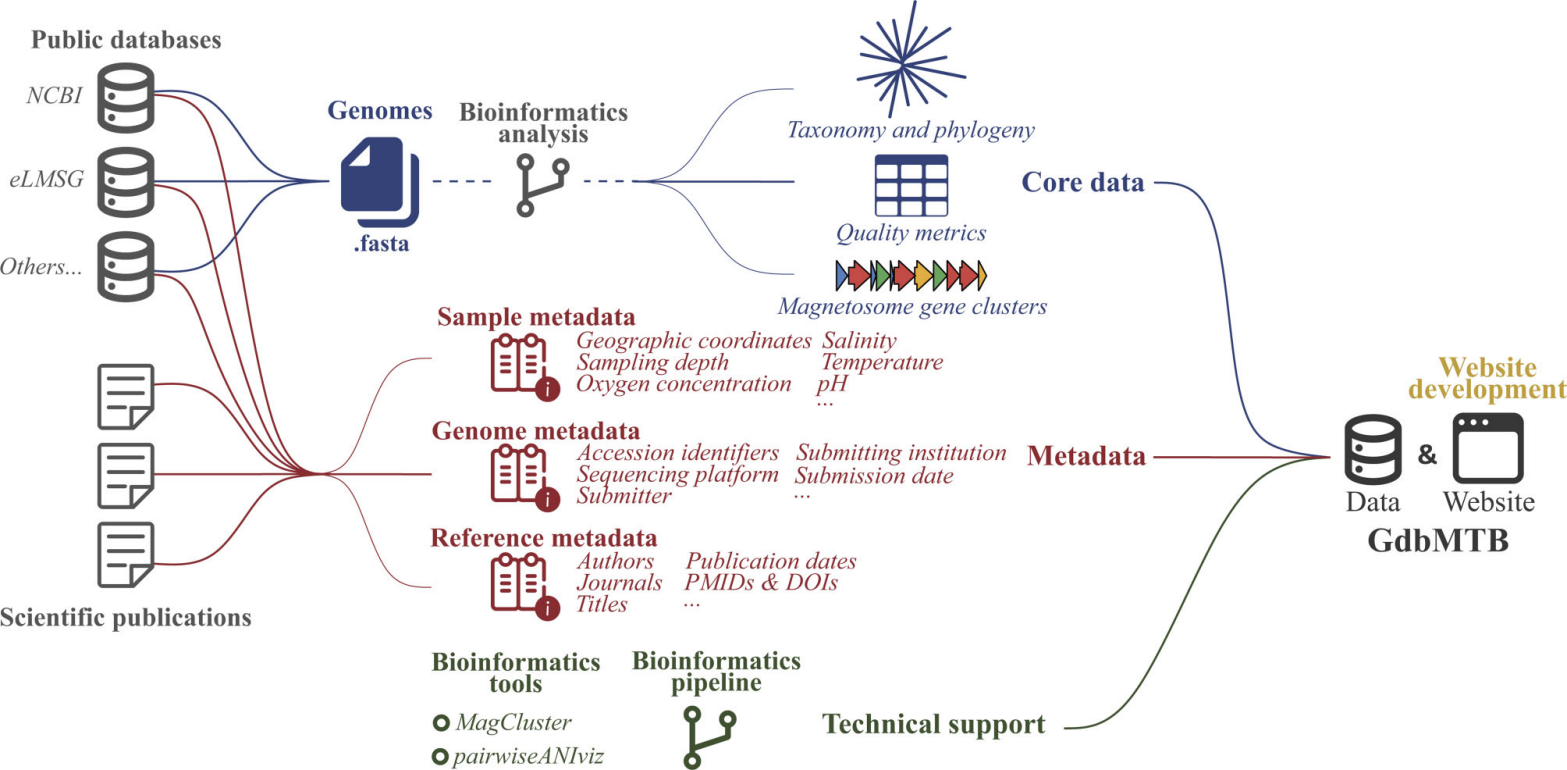
Architecture of GdbMTB. GdbMTB consists of MTB genomic data and a user-friendly website interface. The genomic data has three components: core data, metadata, and technical support. The core data includes MTB genomes sourced from available public databases. Bioinformatics analyses are then applied to profile key features of each genome, including taxonomy, phylogeny, quality metrics, and MGCs. Metadata is collected from both public databases and scientific publications, including three categories: sample metadata, genome metadata, and reference metadata. Sample metadata includes environmental context, such as geographical coordinates and physicochemical factors. Genome metadata contains information related to genome submission, such as various accession identifiers, sequencing platforms, and submitter details. Reference metadata includes publication-related information, such as article titles, authors, PMIDs, and DOIs. Technical support provides bioinformatics tools and a comprehensive analysis pipeline dedicated to MTB research.

GdbMTB is a web-based platform that integrates publicly available genomic data and associated metadata for MTB. The database is organized into three primary categories: core data, metadata, and technical support (Fig. 1). The core data encompasses all publicly reported MTB genomes along with their characteristic information, including genome names, quality metrics, taxonomic classifications, and MGC annotations. The metadata comprises three components: sample metadata (providing environmental context), genome metadata (including accession numbers and submission details), and reference metadata (containing publication-associated information). The technical support offers curated bioinformatics tools and a standardized genomic analysis pipeline to facilitate systematic investigations of MTB phylogeny, ecology, and biomineralization.

### Core data

**Figure 2.**
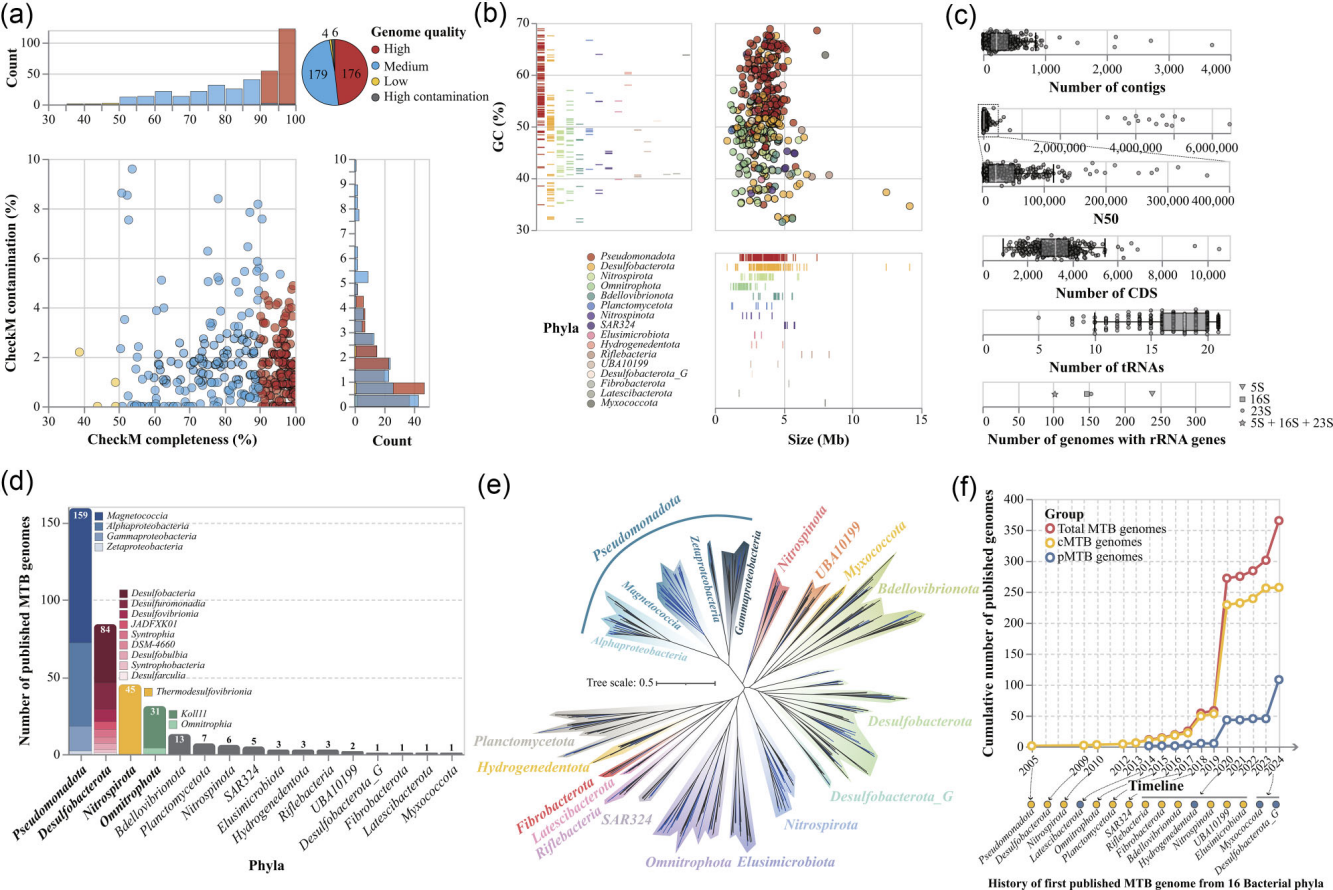
Overview of MTB genomic data in GdbMTB. (a) Genomic quality profile. A unified four-panel composite visualization of the genomic quality of 365 MTB genomes. A shared colour scheme denotes high-quality genomes in red, medium-quality in blue, low-quality in yellow, and high-contamination genomes in grey. The central scatter plot shows CheckM-estimated completeness (%) on the *x*-axis versus contamination (%) on the *y*-axis, with each dot representing an individual MTB genome. The top histogram illustrates the distribution of completeness, while the right histogram displays contamination distribution. A pie chart in the top-right corner summarizes genome counts by quality category: 176 high-quality, 179 medium-quality, 4 low-quality, and 6 high-contamination genomes. (b) Genome size and GC content profile. A composite visualization of the genome size and GC content of 365 MTB genomes, with colours assigned to different bacterial phyla as indicated in the bottom-left legend. The main scatter plot maps genome size (Mb) on the *x*-axis against GC content (%) on the *y*-axis, with each dot representing a single MTB genome. A vertical barcode plot on the left panel shows GC content distribution across phyla, while a horizontal barcode plot on the bottom panel displays genome size distribution across phyla. (c) Other genomic features. Jittered strip charts illustrate the number of contigs, N50 values, number of CDS, and number of tRNA genes for MTB genomes, with each dot representing a genome. Box plots overlay the strip charts, showing the median (white tick), quartiles (box edges), and whiskers (range [Q1–1.5IQR, Q3 + 1.5IQR]). At the bottom, a one-dimensional dot plot shows the number of genomes containing 5S, 16S, 23S, or all rRNA genes, represented by different shapes with shape legend on the right side. (d) Taxonomic distribution of MTB genomes. Bar charts show the number of MTB genomes across different phyla, organized in descending order from left to right. The top four phyla are further divided into class-level stacked bars, arranged by descending genome count from top to bottom, with class names indicated. (e) Species-level phylogenomic tree of MTB. This tree includes 252 species-level representative MTB genomes and 278 non-MTB relatives, built using 120 single-copy marker proteins identified via GTDB-Tk. Branches are coloured by phylum, with phylum names shown. For *Pseudomonadota*, different classes are also labelled. MTB leaves are coloured blue, while non-MTB leaves are black. (f) Cumulative number of published MTB genomes. This line graph tracks the cumulative number of MTB genomes published from 2005 to 2024, with the *x*-axis representing the timeline of publication years and the *y*-axis indicating the cumulative genome count. Three lines are plotted: total MTB genomes (red), confirmed MTB (cMTB) genomes with direct evidence of magnetotactic behaviour or magnetosome biomineralization (yellow), and potential MTB (pMTB) genomes containing MGCs but lacking confirmed magnetosome production (blue). At the bottom, the history of the first published MTB genome for each of 16 bacterial phyla is marked with dashed arrows pointing from the timeline to the phyla, with phyla containing only pMTB genomes coloured blue and those with cMTB genomes yellow.

GdbMTB has compiled 365 publicly available MTB genomes as of June 2024, reported in 47 published studies and preprints between 2005 and 2024 (Fig. 2). All genomes in GdbMTB are accompanied by detailed quality metrics. Assembly quality was evaluated using metrics such as the number of contigs, genome size (bp), GC content (%), and N50 (bp), which provide insights into genome continuity and structural integrity. Gene content was examined by identifying key functional elements, including the number of CDS, transfer RNA (tRNA) genes, and ribosomal RNA (rRNA) genes (5S, 16S, and 23S). Genome completeness and potential contamination were quantified using established tools (see the ‘Materials and methods’ section). These quality metrics collectively ensure that the genomes in GdbMTB are well-characterized, enabling robust and reproducible downstream analyses.

Based on CheckM-estimated completeness and contamination [44], and following the MISAG and MIMAG standards [51], the 365 MTB genomes were classified into four quality categories (Fig. 2a). 176 genomes were categorized as high quality (≥90% completeness and ≤5% contamination), including 13 complete genomes. 179 genomes were classified as medium quality (≥50% completeness and ≤ 10% contamination). Four genomes fell into the low-quality category (<50% completeness and ≤ 10% contamination). Six genomes fell below the standard quality thresholds with high contamination values larger than 10%. These genomes are flagged as ‘high contamination’ to inform users of their limitations in downstream analyses.

The GC content of MTB genomes ranged from 31.5% to 68.9%, with a mean of 51.7% and a standard deviation of 8.6%, exhibiting a lineage-specific pattern distribution (Fig. 2b). In contrast, the genome size distribution was more consistent, with most genomes clustering within the 0.9–6.5 Mb range (Fig. 2b). The 25th, 50th (median), and 75th percentiles were 2.9 Mb, 3.6 Mb, and 4.3 Mb, respectively. However, two genomes exhibited exceptionally large sizes (>10 Mb).

Regarding the rRNA genes, some genomes contain multiple copies of 5S, 16S, and 23S ribosomal RNA genes (Fig. 2c). Specifically, 28 genomes have more than one copy of the 16S rRNA gene, with the highest count found in *Rhodovastum atsumiense* G2-11 (GCA_937425535.1), which contains five copies. For the N50 values, the 365 genomes range from 2222 bp to 6482 810 bp, with an average value of 205 642 bp (Fig. 2c). The number of predicted CDS ranges from 925 to 10 534, with an average value of 3272 CDS (Fig. 2c). The number of tRNA genes spans from 5 to 21 (including genes encoding Selenocysteine (SeC)), with an average of 17 tRNA genes per genome (Fig. 2c).

MTB genomes within GdbMTB are taxonomically affiliated within 16 bacterial phyla, 35 classes, and 49 orders according to the GTDB database version r220 [53] (Fig. 2d). This highlights the broad phylogenetic distribution of magnetotaxis within the bacterial domain. Among these, one genome could not be assigned to any known family, seven genomes could not be assigned to any known genus, and 47 genomes could not be assigned to any recognized species. The *Pseudomonadota* phylum contained the most MTB genomes (159), with *Magnetococcia* as the dominant class (Fig. 2d). The *Desulfobacterota* phylum ranks second in the number of reported MTB genomes, followed by the *Nitrospirota* and *Omnitrophota* phyla. The identification of 13 genomes belonging to the *Bdellovibrionota* phylum suggests potential magnetotaxis ability in predatory bacteria (Fig. 2d) [36, 59].

At the species level, 252 unique taxa were identified using a 95% ANI threshold [60]. The species-level ‘Tree of MTB’ was constructed using representative MTB genomes alongside closely related non-MTB relatives (Fig. 2e), delineating the genetic relationships and lineage divergence of magnetotactic groups.

### Metadata

Genome metadata provides submission details of genomes to public databases, while reference metadata links genomic data to its original research. Together, they enable users to trace data origins, access primary context, thereby enhances transparency, facilitates citation, and supports thorough understanding of the context and associated findings.

Analysis of genome and reference metadata reveals a sustained global effort in MTB research over the past two decades. This is evidenced by the accelerated accumulation of MTB genomes in public databases (Fig. 2f) and the expanding known phylogenetic diversity of magnetotaxis [61], both reflecting growing scientific interest. Contributions are from research groups all over the world. Notable contributions have come from China, the United States, Russia, Germany, Japan, and France.

The continuous growth of the MTB genomic dataset can be attributed to advancements in both research methodologies and sequencing technologies. Most studies rely on magnetic enrichment of MTB cells from environmental samples, while database mining for MGCs has identified novel potential magnetotactic lineages [24, 28, 35, 36, 62]. Although the magnetotactic phenotype remains experimentally uninvestigated for most MGC-containing lineages, these genomes still provide crucial insights into the origin and evolution of MGCs and thus magnetotaxis. A recent study has discovered a silent biosynthetic MGC in a non-MTB alphaproteobacterial strain [63]. As a result, genomes in GdbMTB are classified into two categories: confirmed MTB (cMTB) and potential MTB (pMTB) (Fig. 2f). cMTB genomes are those accompanied by direct evidence of magnetotactic motility (observed via light microscopy or collected via magnetic enrichment) or magnetosome biosynthesis (visualized via electron microscopy). In contrast, pMTB genomes are identified solely by the presence of MGCs, suggesting potential magnetosome biogenesis and magnetotaxis. MGC annotation has revealed pMTB genomes in previously unrecognized bacterial lineages, some of which were later supported by discovery of MTB cells and the corresponding cMTB genomes [25, 26]. Sequencing platforms have evolved from early 454-based [32, 64, 65] and Sanger [66] systems to Illumina platforms (HiSeq [5, 67, 68], MiSeq [69, 70], NovaSeq [22, 30]), which now dominate. More recent studies also employ long-read technologies (PacBio [71, 72], Oxford Nanopore [25, 73]) for higher-quality assemblies.

**Figure 3.**
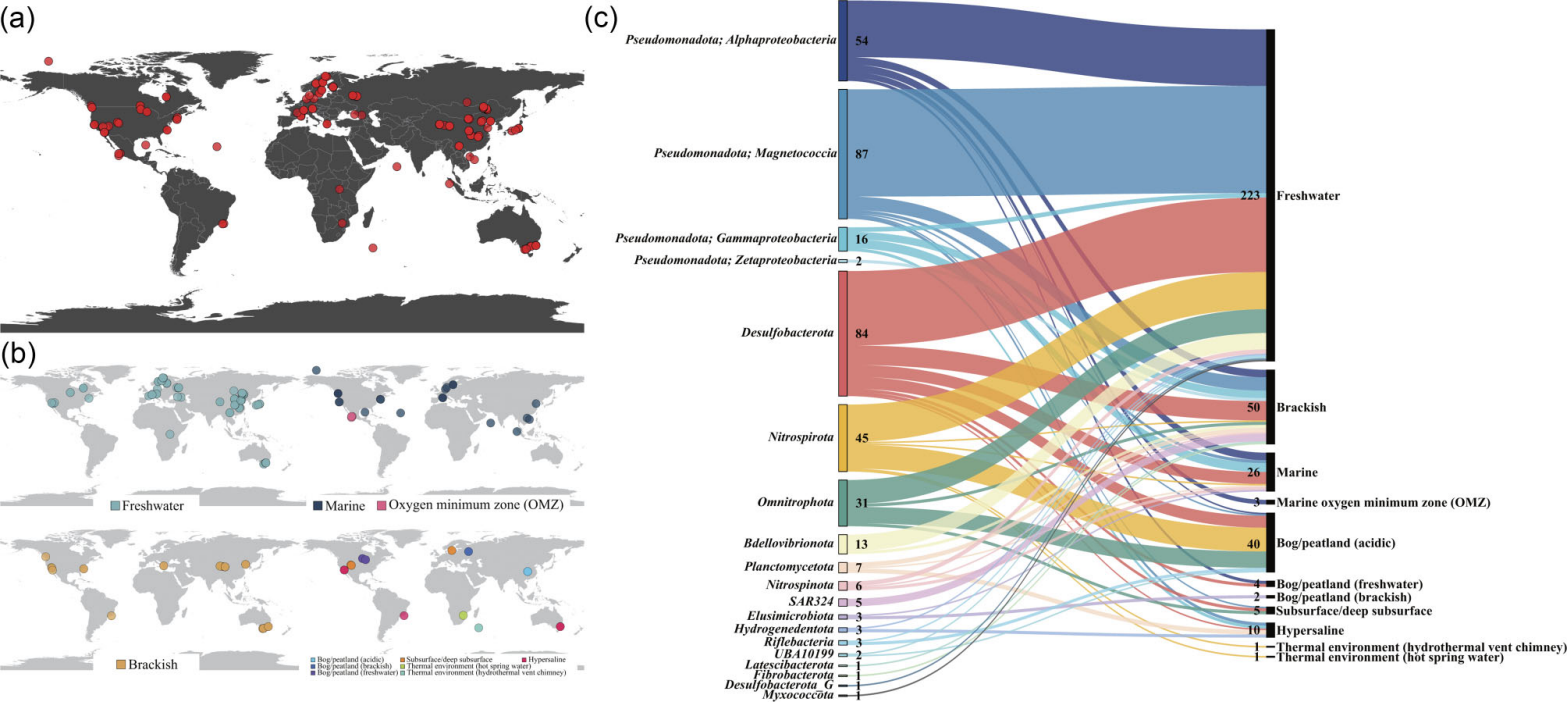
Global distribution and environmental context of MTB genomes. (a) Global distribution of MTB genomes. This figure presents the global geographical distribution of 365 MTB genomes compiled in GdbMTB, with each red dot marking the sampling location of an individual genome. The map highlights the worldwide presence of MTB, with most sampling sites from Asia, Europe, and North America, reflecting the broad geographic scope of the dataset. (b) Habitat-specific distribution. This figure comprises four world maps depicting the geographical distribution of MTB genomes categorized by habitat type, with different colour legends indicated. The first map presents freshwater habitats, the second marine environments and OMZs in the ocean, the third brackish environments, and the fourth employs a multi-colour scheme for other environments. Note that the ‘Brackish’ category is defined based on sampling site descriptions in original publications or a salinity range of 0.5–35 ppt, encompassing both inland salt lakes and estuarine systems. (c) Taxonomic and environmental relationships. A Sankey diagram illustrates the relationships between MTB genomes from different bacterial phyla (left nodes) and their respective environmental types (right nodes). The left nodes, coloured by phylum, are labelled with phylum names and genome counts. The right nodes represent environmental categories, also labelled with names and genome counts. The coloured flows connecting the left and right nodes highlight the complex yet discernible patterns linking MTB taxonomic affiliations to their environmental contexts.

Geographically, GdbMTB currently contains MTB genomes from six continents (except Antarctica) and four oceans (Fig. 3a), with most samples from Asia, Europe, and North America. Among 11 habitat types classified based on the location and physicochemical characteristics of the sampling sites (Fig. 3b), most MTB genomes were obtained from freshwater environments, followed by brackish and marine environments (Fig. 3c). Moreover, previous reports of MTB in various extreme environments [26, 74, 75] (Fig. 3c) suggest the adaptability of MTB to diverse ecological niches.

### Technical support

This section introduces bioinformatics tools developed by our laboratory for MTB research and a recommended MTB genomic analysis pipeline. The tools include MagCluster [58], a MGC annotation tool, and pairwiseANIviz [27], a pairwise ANI visualization tool. MagCluster annotates, screens, and visualizes MGCs, and pairwiseANIviz integrates paired ANI results with GTDB-based classifications, enabling users to evaluate genome novelty more effectively.

**Figure 4.**
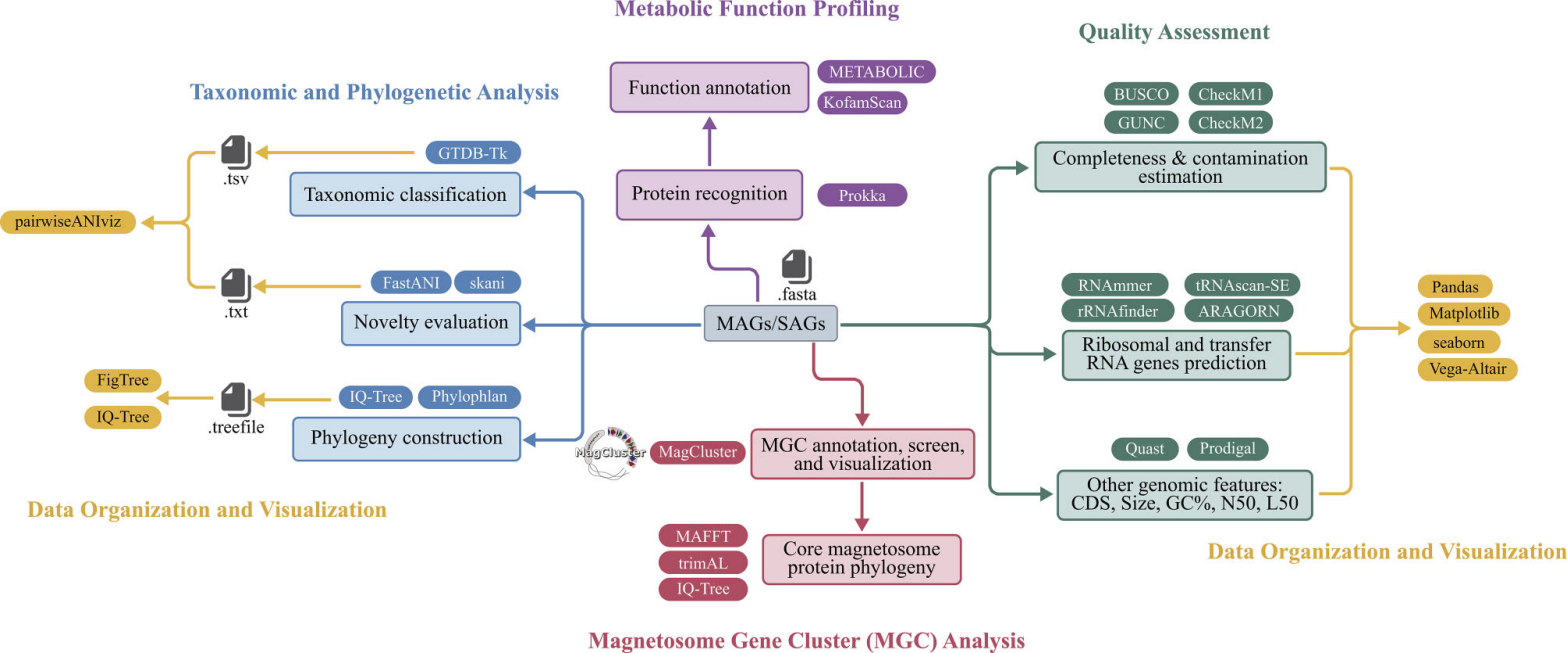
Recommended MTB genomic analysis pipeline with bioinformatic tools. This pipeline serves as a detailed reference for analyzing newly obtained MAGs/SAGs, comprising five components: quality assessment, taxonomic and phylogenetic analysis, MGC analysis, metabolic function profiling, and data organization and visualization. Each component is colour-coded and labelled accordingly. For each section, the associated bioinformatics processes are listed alongside widely accepted tools, with hyperlinks provided.

The recommended MTB genomic analysis pipeline offers a framework for downstream analyses (Fig. 4). It includes quality assessment, taxonomic and phylogenetic analysis, MGC analysis, metabolic function profiling, and data organization and visualization. The pipeline also facilitates comparative genomic analyses, linking genome diversity, metabolic functions, and environmental data to investigate the ecological roles of MTB across diverse taxa and environments.

## Conclusions

GdbMTB represents the first dedicated genomic database for MTB research. By integrating genomic data with rich metadata and analytical tools, GdbMTB offers a robust foundation to explore the fascinating MTB community, particularly those in novel taxa and underexplored habitats. GdbMTB features interactive functionalities that make data exploration intuitive and user-friendly, as well as providing links to external databases for each genome, facilitating exploration of these genomes across multiple databases. Future upgrades of GdbMTB will focus on enhancing online analysis capabilities and will be continuously updated with new data to support ongoing MTB research.

## Data Availability

GdbMTB data, including MTB genomes and associated metadata, has been archived and deposited in Zenodo (DOI: 10.5281/zenodo.14876943) and ScienceDB (DOI: 10.57760/sciencedb.21001).
